# Chronic Intermittent Hypoxia Participates in the Pathogenesis of Atherosclerosis and Perturbs the Formation of Intestinal Microbiota

**DOI:** 10.3389/fcimb.2021.560201

**Published:** 2021-07-01

**Authors:** Chaowei Hu, Pan Wang, Yunyun Yang, Juan Li, Xiaolu Jiao, Huahui Yu, Yongxiang Wei, Jing Li, Yanwen Qin

**Affiliations:** ^1^ Key Laboratory of Upper Airway Dysfunction-related Cardiovascular Diseases, Beijing Anzhen Hospital, Capital Medical University, Beijing Institute of Heart, Lung and Blood Vessel Diseases, Beijing, China; ^2^ Heart Center & Beijing Key Laboratory of Hypertension, Beijing Chaoyang Hospital, Capital Medical University, Beijing, China; ^3^ Key Laboratory of Remodeling-related Cardiovascular Diseases, Beijing Anzhen Hospital, Capital Medical University, Beijing Institute of Heart, Lung and Blood Vessel Diseases, Beijing, China; ^4^ Otolaryngological Department of Beijing Anzhen Hospital, Capital Medical University, Beijing, China

**Keywords:** chronic intermittent hypoxia, obstructive sleep apnea, gut microbiota, atherosclerosis, apolipoprotein E-deficient

## Abstract

Chronic intermittent hypoxia (CIH) is the prominent signature of highly prevalent obstructive sleep apnea (OSA) pathophysiology, which leads to increased risk and aggravation of atherosclerotic cardiovascular diseases. However, whether intestinal microbiota is implicated in the mechanisms linking CIH to arteriosclerosis (AS) pathogenesis remains unclear. The association of CIH with the development of altered gut microbiota (GM) may provide the opportunity to develop preventive strategies for atherosclerotic cardiovascular risk reduction. Animal models of apolipoprotein E-deficient (apoE^-/-^) mice treated with high-fat diet (HFD) and subjected to CIH conditions was applied to mimic the AS observed in patients with OSA. The physiological status and atherosclerotic lesion formation were confirmed by histological analysis. 16S rDNA sequencing of fecal samples was conducted to determine the changes in gut microbial composition. Morphometric analysis demonstrated that CIH caused aggravated atherosclerotic lesions and facilitated AS in apoE^-/-^ mice treated with HFD. The gut bacteria was significantly varied in AS and AS+CIH mice compared with that in the control mice. Significantly perturbed GM profiles were detected in AS mice with and without CIH, with altered microbial α- and β- diversity and shifts in bacterial compositions at phylum and genus levels. While the difference between AS and AS+CIH was observed at different bacteria taxa levels. Aggravation of reduced *Sutterella* and increased *Halomonas*, *Halomonadaceae* and Oceanospirillales was noted in CIH-treated AS mice. The correlation of intestinal bacterial parameters with pathological changes in artery indicated complicated interactions under CIH-induced GM dysbiosis. Furthermore, the gut microbial functions in the potential ability of replication recombination and repair proteins, glycan biosynthesis and metabolism, as well as metabolism of cofactors and vitamins were identified to be further suppressed by CIH. Our findings demonstrated a causal effect of CIH on GM alterations in AS mice and suggested that the disordered GM features in AS development were deteriorated by CIH, which may be associated with AS aggravation. Preventative strategies targeting gut microbiome are highly recommended for intervention of OSA-related AS.

## Introduction

Obstructive sleep apnea (OSA) is the most common sleep disorder, which is characterized by recurrent upper airway collapse during sleep. The prevalence of OSA in 30-70 years old individuals is approximately 27% in males and 11% in females (Whyte and Gibson, 2018). OSA is known to be implicated in metabolic syndromes and cardiovascular diseases, such as arrhythmias, arteriosclerosis (AS), hypertension and acute coronary syndrome (Coughlin et al., 2004; Hedner et al., 2006; Song et al., 2015; Koo et al., 2017; Vizzardi et al., 2017). This condition contributes to significant increase in all causes of mortality. Chronic intermittent hypoxia (CIH) is the primary feature and pathogenic factor associated with OSA pathophysiology and provides a link between OSA and AS (Song et al., 2015; Koehler et al., 2018). It has been suggested that the simultaneous exposure to CIH and high-cholesterol diet aggravates the development of atherosclerotic lesions in animal models (Savransky et al., 2007). However, the mechanisms and key mediators that underlie CIH-associated AS formation remain to be established. The identification of the driving factors governing AS progression under CIH conditions and the exploration of specific strategies targeting this disease have attracted considerable attention.

Gut microbiota (GM) contain a high number of bacteria that colonize the gastrointestinal tract. To date, considerable efforts have been made to uncover the association between gut microbiome and specific diseases, such as hypertension, myocardial infarction, atrial fibrillation, AS and rheumatoid arthritis (Li et al., 2017; Zhou et al., 2018; Yoshida et al., 2018; Zuo et al., 2019; Alpizar-Rodriguez et al., 2019). An increasing number of investigators have focused on the variation of the gut flora in AS and OSA (Karlsson et al., 2012; Tripathi et al., 2018; Ganesh et al., 2018; Ko et al., 2019). The composition and functional capacity of the gut microbiome in association with atherosclerotic cardiovascular disease patients were significantly different from that of healthy control subjects. The enrichment of microbial strains, such as *Enterobacteriaceae* and *Streptococcus* spp. was associated with AS. Similar associations were noted for specific functions, such as metabolism or transport of several molecules important for cardiovascular diseases development (Jie et al., 2017). The presence of specific bacterial taxa has been confirmed to be associated with plasma trimethylamine-N-oxide concentration in the feces of AS patients which is known to be converted from trimethylamine. The latter is formed by intestinal microbiota metabolism of dietary L-carnitine and accelerates the production of AS (Koeth et al., 2013). It is speculated that during the metabolism of cholesterol and lipids, diet and specific GM components affect the development of atherosclerotic plaques (Jonsson and Backhed, 2017).

In CIH-exposed animals, GM composition is disrupted with reduced microbiota species richness, increased abundance of Bacteroidetes and lower Firmicutes (Lucking et al., 2018). Intermittent hypoxia and hypercapnia have also been shown to alter the gut bacterial species from the families of *Mogibacteriaceae*, *Clostridiaceae* in Ldlr^-/-^ and apoE^-/-^ mice. They further may change the levels of microbe-dependent bile acids, enterolignans and fatty acids in Ldlr^-/-^ and apoE^-/-^ mice (Tripathi et al., 2018; Tripathi et al., 2019). In addition, gut microbial dysbiosis of decreased short-chain fatty acid-producing bacteria and increased pathogen number and functional alterations were detected in patients with OSA-hypopnea syndrome (Ko et al., 2019). Despite these findings, data regarding the potential role of fecal microbiota in the induction of CIH and AS are limited.

The present study aimed to reveal the shifts of GM in response to CIH induction during the development of AS and provide further evidence to develop preventive strategies for atherosclerotic cardiovascular risk reduction in patients suffering from OSA. ApoE^-/-^ mice fed with HFD were exposed to CIH, mimicking atherosclerotic cardiovascular diseases. 16S rRNA amplicon profiling was performed to examine the changes in bacterial composition and functions. Altered microbiota diversity and altered abundance of specific microbes in AS mice were shown to be modulated by CIH.

## Materials and Methods

### Animal Model

All apoE^-/-^ mice were established from C57BL/6 mice. All mice were housed in a specific pathogen-free animal facility at the key laboratory of upper airway dysfunction-related cardiovascular diseases. The animals were maintained on a 12:12 h day:night cycle with constant access to food and water. 8-week-old male mice were randomly divided into three groups as follows: wild-type (WT) mice in the control group were fed with chow diet, whereas apoE^-/-^ mice in the AS group were fed with HFD(containing 21% fat and 0.15% cholesterol, Beijing Huafukang Biotechnology Co) under normoxic conditions. ApoE^-/-^ mice in the AS+CIH group were fed with HFD and subjected to CIH (5% O_2_ at nadir, 20 cycles∙hour^−1^) for 12 weeks. For the induction of CIH, mice were raised in customized standard cages with a gas control system regulating the room airflow (N_2_ and O_2_) (Oxycycler A84, US). The system programs enabled the adjustment of the fraction of inspired O_2_ from 20.9% to 5% over a 120 seconds period, following a rapid reoxygenation to 20.9% O_2_ in the subsequent 60 seconds. Hypoxia occurred once per 180 seconds throughout the 12-h dark time. Under the 12-h light time, the CIH mice were maintained under normoxic environmental conditions. Control mice were exposed to normoxic conditions. All experiments were performed according to the guidelines for animal experiments reported at capital medical university.

### Atherosclerotic Lesion Assessments

Atherosclerotic lesions were assessed by Oil Red O stainings. The adventitial fat of the aortas was thoroughly trimmed away. The aortas were fixed in 4% paraformaldehyde over 24 h and washed in PBS twice. Subsequently, the aortas were dissected longitudinally with a scalper and stained with Oil Red O for 1 h at 37°C. The fat plaques were differentiated into orange or bright red in 75% ethanol, whereas other parts were nearly colorless. The aortas were washed twice with distilled water. To assess the atherosclerotic lesion size at the aortic sinus, the ascending aorta samples containing the aortic sinus were fixed with 4% paraformaldehyde and embedded in paraffin. Subsequently, the sectioned samples were stained with Oil Red O. The stained sections were digitally captured and the Oil Red O-positive and lesion areas were measured using the ImageJ software (n=4 for WT, and n=5 for AS and n=4 for AS+CIH).

### Masson Staining

The aortic roots were immersed in 4% paraformaldehyde. The fixed tissues were dehydrated, cleared and embedded in paraffin waxes. The paraffin blocks were sectioned and stained with masson trichrome to determine the area of collagen and severity of fibrosis. Stained sections were digitally captured and the percentage of the stained area (the stained area per total atherosclerotic lesion area) was calculated. n=4 for WT, and n=5 for AS and n=5 for AS+CIH.

### Bacterial 16S rRNA Sequencing, Annotation and Kyoto Encyclopedia of Genes and Genomes (KEGG) Function Profiles

Based on the samples’ unique barcode, raw reads were assigned to different samples and the assigned paired-end reads of each sample were merged to raw tags by FLASH. The merged raw tags were filtered and developed into clean tags according to QIIME (Version 1.7.0.) quality controlled processes. Subsequently, the clean tags were aligned to the Gold database and the chimera sequence was detected using the UCHIME algorithm. The non-chimera clean tags were defined as effective tags. The effective tags were clustered into operational taxonomic units (OTUs) with ≥ 97% similarity as determined by Uparse. The representative sequence for each OTU was selected and the taxonomic information was annotated using RDP classifier and the GreenGene database. The sequences which could not be assigned into a specific taxonomy level were labeled as “unclassified”. Phylogenetic investigation of communities was performed by the Reconstruction of Unobserved States (PICRUSt) version 1.0.0 to generate the KEGG ontology profiles. The animal version was used to assign ontology into the KEGG pathway at the module level (n=7 for WT, and n=8 for AS and AS+CIH).

### Statistical Analysis

Categorical data were compared with the Fisher’s exact test. For comparisons between two independent groups, an unpaired Student’s t test or a Mann-Whitney U test was used. For comparison of three groups or more, one-way analysis of variance with Tukey’s *post hoc* analysis was used or a Kruskal-Wallis test followed by a Dunn’s test was employed. Correlation analyses were performed based on Spearman’s correlation. The QIIME software (Version 1.7.0.) package was used to analyze the α- and β- diversity. For the determination of the α-diversity, the Chao1 index, Shannon index, Ace, invsimpson and simpson were calculated based on the genera profile of WT, AS and AS+CIH mice. Subsequently, the determination of the β-diversity was achieved using an OTU table to generate the Bray-curtis distance. The unweighted and weighted principal coordination analysis (PCoA) and non-metric dimensional scaling (NMDS) were performed and displayed by the WGCNA package, extra font package and ggplot2 package in the R software. The differential abundance of genera was tested by the Wilcoxon rank sum test and the *P* values were corrected for multiple testing with the Benjamin and Hochberg method. The Linear discriminant analysis (LDA) Effect Size (LEfSe) version 1.0 was used to identify taxa and KEGG pathways, which were significantly different among groups (LDA score (log10) = 2 as cutoff value).

## Results

### CIH Exacerbates the Atherosclerotic Lesions in AS Mice

**Figure 1:**
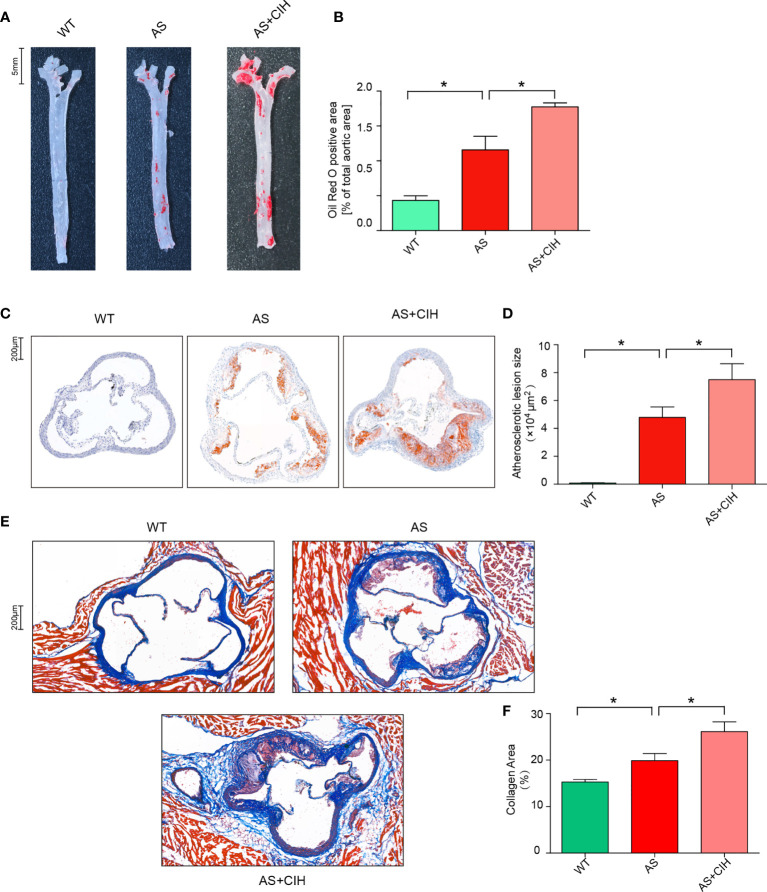
CIH induces aggravation of atherosclerotic lesion burden in HFD-fed apoE^-/-^ mice. **(A, B)** Representative photomicrographs of Oil Red O stainings and quantitative analysis of Oil Red O-positive area in the whole aortas. n=4 for WT, and n=5 for AS and n=4 for AS+CIH. Scale bar represents 5 mm. **(C, D)** Representative photomicrographs of Oil Red O stainings and quantitative analysis of atherosclerotic lesion area in the aortic sinus. n=4 for WT, and n=5 for AS and n=4 for AS+CIH. Scale bar represents 200 μm. **(E–F)** The degree of fibrosis in the aortic sinus was determined in masson-stained cross-sections. Representative sections and quantitative analyses of collagen area are shown. n=4 for WT, and n=5 for AS and n=5 for AS+CIH. Scale bar represents 200 μm. *P value < 0.05, Kruskal-Wallis.

To determine the contribution of CIH in the development of AS, 8-week-old male apoE^-/-^ mice were fed with HFD (AS mice model) and exposed to a CIH environment (5% O_2_ at nadir, 20 cycles∙hour^−1^) for 12 weeks. Oil Red O stainings of the aortas were performed and lipid deposition was measured. As shown in **Figures 1A, B**, the aortic atherosclerotic lesion burden was significantly enhanced in AS mice, whereas it was deteriorated under CIH conditions. In addition, mice treated with CIH exhibited significantly increased lesion size in the aortic root compared with that noted in AS mice (**Figures 1C, D**). Furthermore, increased levels of collagen and aggravated fibrosis were detected in the aortic sinus of CIH-treated AS mice as determined by immunohistochemical studies (**Figures 1E, F**). These findings suggested that CIH exposure promoted the stability of atherosclerotic plaques and facilitated the development of AS.

### Altered Microbiota Diversity Correlates With CIH Exposure in apoE^-/-^ Mice Fed With HFD

**Figure 2:**
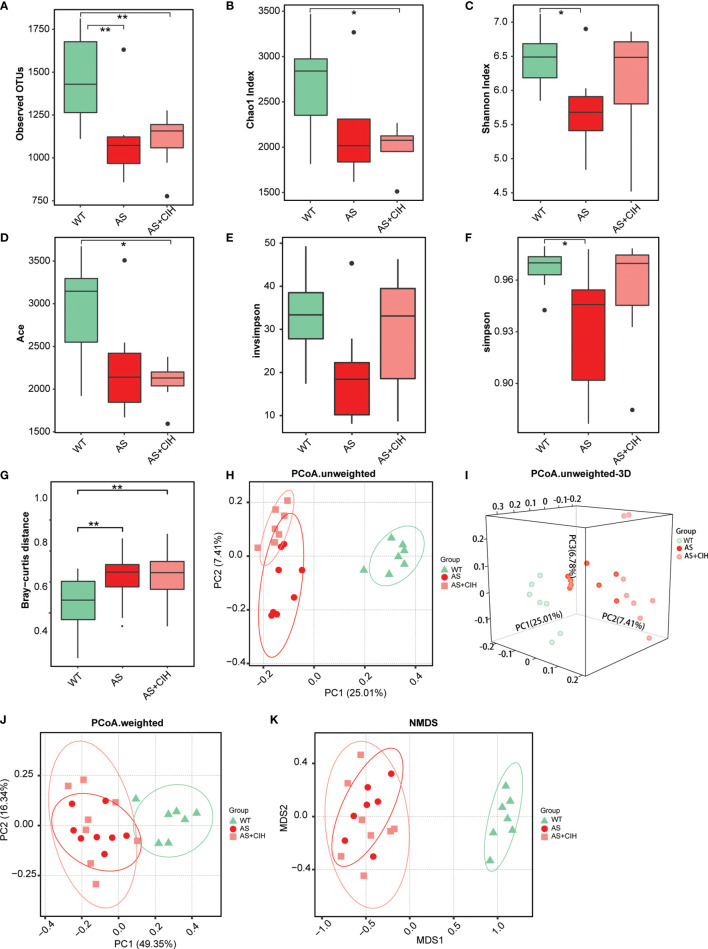
Gut microbial diversity within-sample (α) and between-sample (β) in CIH treated apoE^-/-^ mice fed with HFD. **(A)** Number of observed OTUs in control (WT), AS and atherosclerotic mice with CIH (AS+CIH). b-f. Parameters of α-diversity at the genus level, including Chao index **(B)**, Shannon index **(C)**, Ace **(D)**, invsimpson **(E)** and simpson **(F)** among groups. **(G)** Bray-curtis distance, parameters of β-diversity in the WT, AS and AS+CIH groups. The boxes represent the inter quartile ranges, whereas the inside line represent the median and the points are outliers. *P value < 0.05, **P value < 0.01, Kruskal-Wallis. **(H, J)** Unweighted **(H)** and weighted **(J)** unifrac PCoA plots of samples from groups at the genus level. **(I)** 3D version of the unweighted unifrac PCoA plots. **(K)** NMDS plots of each sample based on abundances of the genera. n=7 for WT, and n=8 for AS and AS+CIH.

To investigate the global shifts of gut flora following CIH induction, the 16S rRNA V3-V4 regions of the fecal samples from HFD-fed apoE^-/-^ mice with or without CIH treatment were amplified and sequenced. A total of 1,463,268 qualified tags from 1,831,889 raw tags were filtered and a total of 99.4% of all qualified tags were clustered into qualified OTUs. Finally 7,130 qualified OTUs were obtained for analysis. For the overall richness of gut microbiome, the bacterial load was decreased significantly in atherosclerotic mice compared with that of the WT group as indicated by the observed OTUs (**Figure 2A**). The analysis of α-diversity (within-sample) indicated notably reduced Shannon diversity and simpson index in the atherosclerotic mice compared to those of the WT, whereas much lower Chao and Ace indices were noted following CIH induction (**Figures 2B–F**). In contrast to these findings, no significant changes was noted in bacterial α-diversity between animals, which underwent CIH or not. In addition, the bacterial β-diversity (between-sample) was perturbed following CIH induction. An apparent difference was noted in the Bray-curtis distance between microbiota in both groups of atherosclerotic mice, although the impact of CIH on AS mice was relatively small (**Figure 2G**). The PCoA was assessed on the basis of weighted and un-weighted unifrac distances and the data illustrated significant differences and separations among WT, AS and CIH-treated mice (**Figures 2H–J**). Notably, a clear cluster was noted in the un-weighted distance between the samples from the AS and WT group (**Figures 2H, I**). Similar trends in the β-diversity parameters were demonstrated by NMDS analysis (**Figure 2K**). Gut microbiota diversity analysis indicated that CIH exposure played a significant role for the disordered microbiome in AS.

### CIH Affects the Global GM Composition of Atherosclerotic Mice at the Phylum and Genus Level

**Figure 3:**
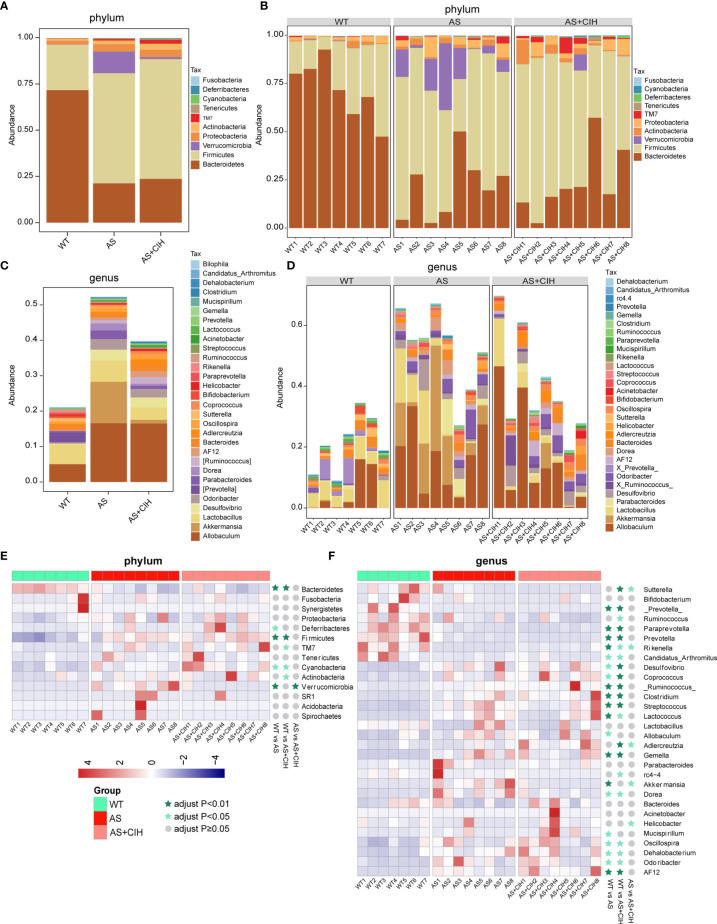
Gut taxonomic profiling of bacterial communities following CIH induction. **(A–C)** The bar plots indicate the mean relative abundance of top 10 phyla **(A)** and the top 30 genera **(C)** annotated in WT, AS and AS+CIH groups, where phyla and genera are differentiated by color. **(B, D)** Bar plots of the relative abundance of the top 10 phyla **(B)** and top 30 genera **(D)** annotated in each sample from WT, AS and AS+CIH. Phyla and genera are differentiated by color. **(E, F)** Heat map showing the top 14 **(E)** and top 30 **(F)** dominant bacteria annotated at the phyla and genus level respectively. The relative abundances are expressed by Z scores by subtracting the average abundance and dividing the standard deviation of all samples. Z scores are represented by blue color when the row abundance is lower than the mean and with red when the row abundance is higher than the mean. The taxon at *P* value <0.01 is marked with dark green star, whereas when the *P* value <0.05 the taxon is marked with light green star, and when the *P* value ≥0.05 with gray circle. n=7 for WT, and n=8 for AS and AS+CIH.

The observed OTUs in the fecal samples were annotated to the corresponding bacteria taxa, and 99.83% and 38.76% of all reads were assigned into phyla and genera, respectively. To track CIH-induced global taxonomic shifts in microbial composition, we evaluated the dominant bacteria phyla and genera in CIH-induced mice. Taxonomic annotation and abundance profiling was carried out in **Figure 3**. It was found that Bacteroidetes, Firmicutes, Actinobacteria and Proteobacteria were the most abundant phyla in the gut environment of CIH-induced atherosclerotic mice (**Figures 3A, B**). Similar alterations including reduced abundance of Bacteroidetes and increased Firmicutes were detected in atherosclerotic mice in the presence or absence of CIH. It was shown that CIH promoted a stronger increase in the abundance of Actinobacteria and TM7. At the genus level, the top 30 genera that colonized the intestine of CIH mice were identified. *Allobaculum*, *Lactobacillus*, *Desulfovibrio*, *Oscillospira*, *Adlercreutzia*, *Bacteroides*, *Helicobacter* and *Odoribacter* were the most abundant bacteria following CIH induction (**Figures 3C, D**). Heat-map in **Figures 3E, F** showed relative abundance of the top14 most dominant phyla and top 30 genera annotated, and their distribution in each sample. The majority of the bacteria enriched in WT were deficient in AS and CIH-induced mice, whereas the dominant microbiota in CIH mice, such as Firmicutes, *Oscillospira* and *Lactococcus* were diminished in the WT group. Similar results were noted at the phylum and genus levels in atherosclerotic mice both in the presence and absence of CIH compared with the WT group. The impact of CIH on gut microbial profiles was paralleled with the alterations during AS, at least in the microbial diversity and taxonomic composition.

### CIH Alters the Intestinal Flora in Atherosclerotic Mice

**Figure 4:**
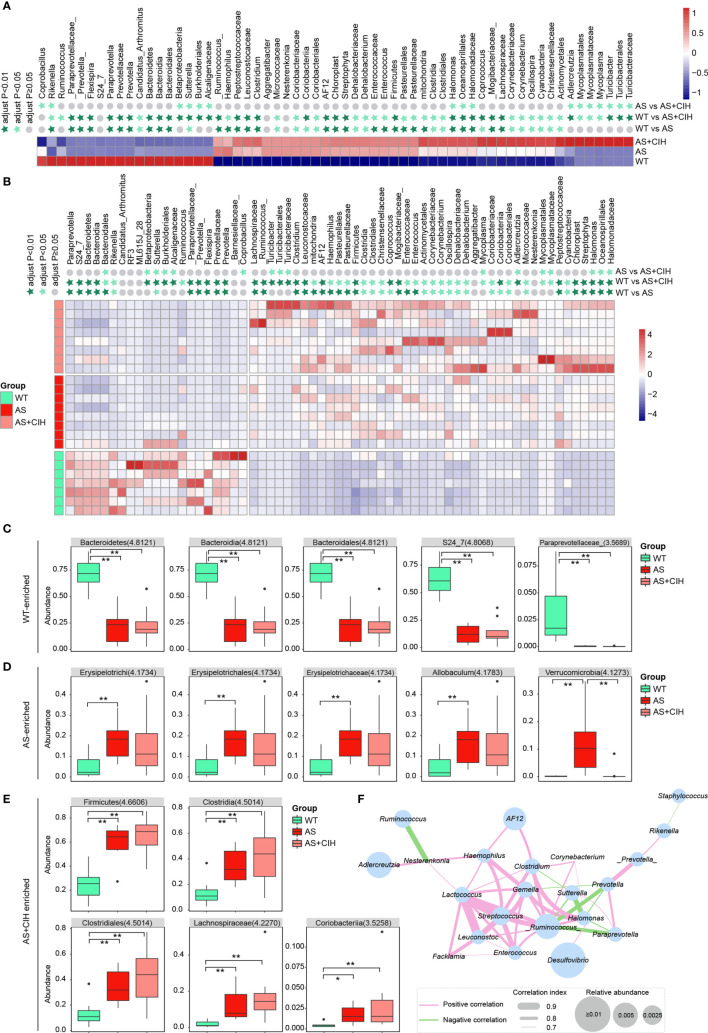
Comparison of the bacteria potentially distinct among WT, AS and AS+CIH groups. **(A, B)** Relative abundances of the top 18 most significantly decreased bacteria and 43 most strikingly enriched taxon in AS+CIH. The taxa shown are statistically significant when all the three groups are included in the calculation. Panel a corresponds to the mean expression noted in the different groups and panel b is representative of the mean expression noted in the samples. The abundance profiles are transformed into the Z scores by subtracting the average abundance and dividing the standard deviation of all samples. The Z score is a negative blue indication when the row abundance is lower than the mean and a red indication when it is higher. The taxon at *P* value <0.01 is marked with dark green star, whereas when the *P* value <0.05 with light green star and when the *P* value ≥0.05 with gray circle. **(C–E)** Box plots showing relative abundance of the top 5 WT-enriched **(C)**, AS-enriched **(D)** and AS+CIH-enriched **(E)** bacteria, which varies significantly across the three groups. The boxes represent the inter quartile ranges, the lines inside the boxes denote medians and circles are outliers. The numbers in brackets indicate the LDA score in LEfSe analysis. *P value < 0.05, **P value <0.01. **(F)** Co-occurrence network of the significantly different genera among groups. The thresholds derived from the Spearman’s correlation analyses are r≥0.7 and *P ≤* 0.05. The pink lines connecting two genera indicate positive correlation and the green lines represent negative correlation. The thickness of the line is proportional to the value of correlation and the size of the nodes is proportional to their relative abundance. n=7 for WT, and n=8 for AS and AS+CIH.

To determine whether CIH cause significant changes in the profiles of intestinal microbiota, statistical analysis was performed and comparison of the bacteria potentially distinct among WT, AS and AS+CIH groups was preliminarily performed in **Figure 4**. The LEfSe analysis coupled with Kruskal-Wallis and Wilcoxon tests were applied and bacteria with *P*<0.05 and LDA score higher than 2 were considered to have undergone significant variations. The taxa shown were statistically significant when all the three groups were included in the calculation. A considerably higher abundance of *Rikenella*, Bacteroidetes and Bacteroidia was noted in the control group, while gut microbes such as *Clostridium*, Clostridia, *Coprococcus*, *Enterococcus* and Actinomycetales were significantly enriched in the AS and CIH-induced mice (**Figures 4A, B**). It was notable that the majority of these bacteria that accumulated in the CIH-induced mice indicated dynamic dysbiosis and an increased trend of change from the WT group to the AS and CIH-induced AS state. The increase noted in the enrichment of *Halomonadaceae*, *Lachnospiraceae*, *Corynebacteriaceae*, *Clostridium*, Clostridia, Clostridiales, *Coprococcus*, *Corynebacterium* and *Oscillospira* was gradually deviated from the steady state in the WT group to the atherosclerotic group and the data indicated the most drastic augmentation following CIH induction (**Figures 4A, B**). It was shown that the impairment of the gut ecosystem during the development of AS was extremely deteriorated following CIH induction.

**Figures 4C–E** show relative abundance of the top 5 WT-enriched, AS-enriched and AS+CIH enriched bacteria statistically significant when all the three groups were included in the calculation. The statistical significance between WT and AS, WT and AS+CIH, AS and AS+CIH was further marked. The assessment of the gut flora markers among different groups demonstrated that Bacteroidetes, Bacteroidia, Bacteroidales, S24_7, and *Paraprevotellaceae* were the most abundant taxon noted in the control mice, while Erysipelotrichi, *Erysipelotrichaceae*, *Allobaculum*, Erysipelotrichales, and Verrucomicrobia were the most dominant bacteria in the atherosclerotic mice (**Figures 4C, D**). The high abundance of Firmicutes, Clostridia, Clostridiales, *Lachnospiraceae* and Coriobacteriia were the key features noted in the gut microbiome following CIH induction (**Figure 4E**). These identified microbiota exhibited similar patterns of change in atherosclerotic mice in the presence and/or absence of CIH induction, with the exception of Verrucomicrobia. Subsequently, the association among the gut genera was significantly affected by CIH. A co-occurrence network was obtained based on Spearman’s correlation analysis and the genera with r≥0.7 and *P ≤* 0.05 were shown in **Figure 4F**. *Desulfovibrio* was the most abundant genera within the network, which indicated slightly positive correlation with *Halomonas*. While *Halomonas* was directly associated with *Ruminococcus* and *Clostridium*. *Ruminococcus*, *Lactococcus* and *Streptococcus* were the core genera in the network, with extremely high correlation noted with the other genera, such as *Enterococcus* and *Haemophilus*. These key gut microbes may play key roles in the development of AS under CIH conditions through complex interactions with each other.

Moreover, to further investigate the features more likely to explain the differences between groups, whether there were specific bacteria statistically significant between WT and AS, AS and AS+CIH was shown in **Figure S1**. The Bacteroidetes, Cyanobacteria and Firmicutes phyla and 35 genera, including *Akkermansia*, *Desulfovibrio*, *Lactobacillus* were regarded as the most significant bacteria, which were distinguished in the gut microbiome of AS from that of the control mice (**Figures S1A, B**). Verrucomicrobia, *Adlercreutzia* and *Akkermansia* contributed to the differences noted between AS and CIH-coupled AS groups (**Figures S1C, D**). Certain shifts were noted in gut microbes, such as decreased *Adlercreutzia*, *Helicobacter* and increased *Staphyiococcus* and *Akkermansia* that were specific in the AS group. However, the reduction of *Sutterella* and the increase in the abundance of *Halomonas* were noted in the atherosclerotic mice following CIH induction. Taken together, much similarity of AS and AS+CIH as compared with WT was identified, and the difference between AS and AS+CIH was further focused on.

**Figure 5:**
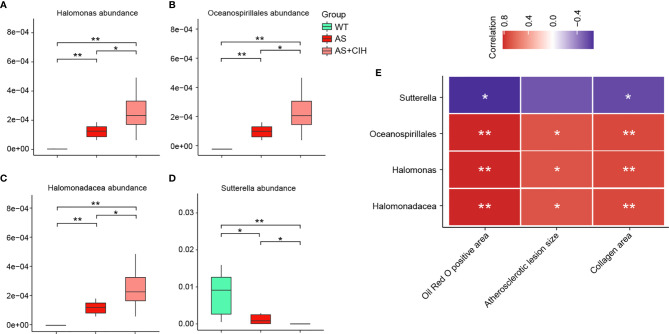
Correlation of pathological changes in artery with the specific gut bacteria further affected by CIH in AS mice. **(A–D)** Box plots showing relative abundance of specific bacteria significantly altered in AS mice and further influenced by CIH. The boxes represent the inter quartile ranges, whereas the inside line represent the median and the points are outliers. *P value < 0.05, **P value < 0.01, Kruskal-Wallis. **(E)** Correlation analysis of *Halomonas*, *Halomonadaceae*, Oceanospirillales and *Sutterella* with parameters of pathological changes in artery, including Oil Red O positive area, atherosclerotic lesion size and collagen area. The thresholds derived from the Spearman correlation analyses were r≥0.5 and *P ≤* 0.05. *P value <0.05, **P value <0.01.

For bacterial taxa significantly increased or decreased in AS mice as compared with controls, *Halomonas*, *Halomonadaceae* and Oceanospirillales were further enhanced by CIH, and *Sutterella* was further suppressed in the AS+CIH group. The relative abundance of these focused bacteria was shown in **Figures 5A–D**. Moreover, the abundance of these CIH-responsive bacteria was dramatically correlated to the pathological changes in artery, including Oil Red O positive area, atherosclerotic lesion size and collagen area (**Figure 5E**). In detail, *Halomonas*, *Halomonadaceae* and Oceanospirillales were positively linked to the severity of atherosclerotic lesions and fibrosis in AS mice, whereas *Sutterella* was observed to be negative correlated with Oil Red O positive area and collagen area. The correlation of intestinal bacterial parameters with pathological changes in artery indicated complicated interactions under CIH-induced GM dysbiosis.

### The Gut Microbial Functions in Atherosclerotic Mice Are Altered Following CIH Induction

**Figure 6:**
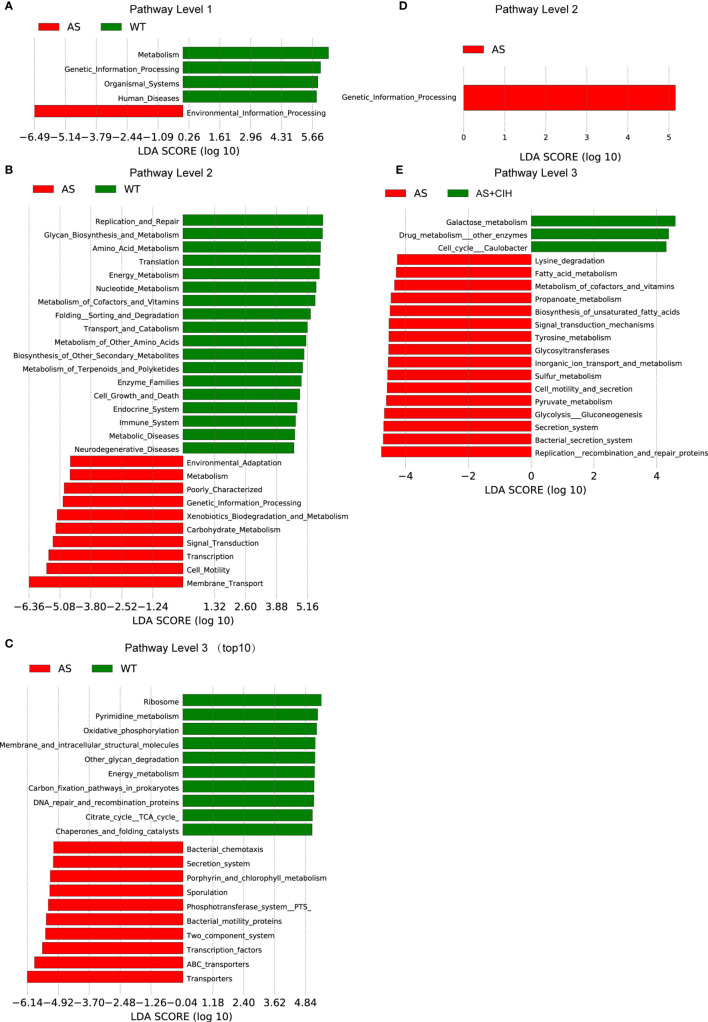
Gut microbial functions in atherosclerotic mice changed following CIH. **(A–C)** PICRUSt shows that KEGG pathways were significantly different between AS (red) and control (green) mice with regard to the pathway level 1 **(A)**, 2 **(B)** and 3 **(C)**. **(D, E)** Bar plots of KEGG pathways significantly different between AS mice (red) and the mice housed under CIH conditions (green). n=7 for WT, and n=8 for AS and AS+CIH.

OTUs were aligned into the reference database from PICRUSt. A total of 5,053 KEGG orthologs were parsed and 258 KEGG pathways were mapped into. LEfSe analysis suggested that 124 pathways were significantly different between the AS and the control groups, whereas 19 pathways varied in the CIH group (*P*<0.05, LDA score>4). KEGG pathway analysis demonstrated that microbial functions associated with glycan biosynthesis and metabolism, metabolism of amino acids, nucleotides, cofactors, vitamins, and pyrimidines were significantly deficient in atherosclerotic mice (**Figures 6A–C**). The functions of transporters, membrane transport, carbohydrate metabolism and bacteria motility proteins, which were enriched in the AS group may participate in the alterations observed in host metabolic profiles during the development of disease. Under CIH conditions, we found microbial functions of replication recombination and repair proteins, glycan biosynthesis and metabolism, as well as metabolism of cofactors and vitamins, which were significantly deficient in AS mice, further suppressed by CIH (**Figures 6D, E**).

## Discussion

In the present study, the CIH-related shifts of intestinal microbiota were confirmed to be associated with AS. A controlled diet by feeding both apoE-/- and WT mice on the same high-fat diet, would result in a comparison between AS and obesity group instead, as WT mice on high-fat diet have been widely used as obesity model previously (de La Serre et al., 2010; Hariri and Thibault, 2010). The comparison between WT (chow diet) and apoE-/- mice (high-fat diet) is actually comparing healthy control and AS group, which has also been performed by other investigators previously (﻿Liu et al., 2019; Cao et al., 2020). Thus, in the present study, WT mice fed on chow diet were considered as healthy control group, and apoE-/- mice on high-fat diet overall were applied to imitate atherosclerotic disease. The data indicated the presence of aggravated atherosclerotic lesion formation in AS-prone mice following CIH induction. Significant distinctions in global GM structures, such as bacterial α-diversity and β-diversity, were detected in AS-prone mice in the presence and/or absence of CIH. The intestinal microbiota in AS mice were dramatically altered compared with that of the control animals, and CIH induced bacterial dysbiosis and alterations in AS mice lead to a more deteriorated profile. We further observed a disturbance in bacterial functions caused by CIH. The hypoxia-sensitive bacteria in the gut may be linked to the formation of atherosclerotic plaque and AS in OSA patients.

The GM profiles have been previously shown to be significantly associated with symptomatic AS in both human patients and experimental mice (Karlsson et al., 2012; Jie et al., 2017; Brandsma et al., 2019). Previous investigations have suggested that reduced gut bacterial α-diversity is positively correlated with carotid-femoral pulse wave velocity and arterial stiffness (Menni et al., 2018), which is one of the earliest markers of arterial remodeling (Kim and Kim, 2019). In addition, CIH is known to lower the fecal microbiota species richness as determined by the Chao1 index and the total number of observed species, but not by the simpson and Shannon indices (Lucking et al., 2018). Consistent with these observations, the data demonstrated that in the AS mouse model, only the α-diversity indices including observed OTUs and Shannon and simpson indices were prominently decreased. However, following the induction of CIH, the atherosclerotic mice showed suppressed microbial richness by the Chao and Ace indices, instead of the Shannon and simpson indices. These findings thus indicated that although reduced GM diversity has been known to be implicated in both CIH and AS, different microbial indices varied across CIH and AS. The Shannon and simpson indices were more likely to decrease during the development of AS, while Chao and Ace were more sensitive in CIH-induced AS. Despite these findings, synergistic alterations in bacterial β-diversity were detected following exacerbation of AS by CIH, with a more strong trend towarding separation of microbiota communities from the controls.

Since the fecal microbiota structures of AS mice were disturbed by CIH, we focused in more detail on the bacterial taxa. In the present study, *Halomonas*, Oceanospirillales and *Halomonadaceae* were shown to be increased in the gut of AS mice and were significantly increased following CIH induction. Recently it has been reported that genera of intestinal bacteria annotated as pathogenic microorganisms such as *Halomonas* is quite predominant in the gut of infected zebrafish (Yang et al., 2017; Qian et al., 2019). It is interesting to note that the growth of *Halomonas* is also correlated to periodontal health (Park et al., 2015). *Halomonas*-rich salivary microbiome was suggested to be significant indicators of high IL-1β (Acharya et al., 2017). In fact, the abundance of *Halomonas* has been previously believed to has pathogenic potential and cause infections and contamination (Stevens et al., 2009). Increased abundance of Oceanospirillales was identified to be significantly associated with disease and discriminate healthy and diseased individuals (Hoj et al., 2018). The enrichment of these bacteria was further increased by CIH induction in AS mice. These bacterial species were suggested to be potential players in mediating the development during OSA-linked atherogenesis.

In contrast to these findings, the data indicated that the abundance of *Sutterella* in the gut of AS mice was significantly reduced by CIH. *Sutterella* has been widely considered to exert protective immunoregulatory profile *in vitro*, and is correlated with better outcomes in inflammatory bowel disease patients (Morgan and Harris, 2015; Hiippala et al., 2016; Berer et al., 2017). Lower abundance of *Sutterella* was frequently observed in various diseases such as Crohn’s disease, nonalcoholic steatohepatitis and multiple sclerosis (Gevers et al., 2014; Del Chierico et al., 2017; Berer et al., 2017; Schepici et al., 2019). And butyrate is demonstrated to protect against non-alcoholic steatohepatitis by promoting the enrichment of promising probiotic genera *Sutterella* (Ye et al., 2018). In addition, multiple sclerosis patients on disease-modifying therapy showed improved abundance of *Sutterella* (Jangi et al., 2016). Reduced *Sutterella* was also reported in patients suffering Type 2 diabetes and restored when receiving metformin or Roux-en-Y gastric bypass for therapy (Wang et al., 2018; Wang et al., 2020). Therefore, it was shown that *Sutterella* may protect against metabolic disorders and cardiovascular diseases. It may be assumed that CIH exerts a role in AS development by lowering *Sutterella* abundance in the intestine.

The present study further showed that the microbial functions of replication recombination and repair proteins, glycan biosynthesis and metabolism, as well as metabolism of cofactors and vitamins were significantly suppressed in AS mice, and further aggravated by CIH. These metabolic pathways have been revealed to be relevant to AS formation. In patients with hyperhomocysteinemia, the suppression of essential cofactors such as B-vitamins folate and vitamin B12 was observed (Schroecksnadel et al., 2005). And nutritional deficiency in vitamin cofactors of folate, vitamin B6, B12, and B2 was demonstrated to be responsible for hyperhomocysteinemia (Selhub et al., 1993; Franken et al., 1994; Mastroianni et al., 2000), which subsequently contributes to endothelial dysfunction and accelerates AS (Lawrence de Koning et al., 2003). Additionally, previous study has demonstrated long-term poor vitamin K nutritional status as an independent risk factor for AS (Berkner and Runge, 2004; Vermeer and Theuwissen, 2011). Moreover, it was recently shown that metabolic responses of gluconeogenesis was inhibited with acute hypoxic stress and recovered following reoxygenation (Sun et al., 2020). Gluconeogenesis is involved in flavin monooxygenase 3-promoted dyslipidemia and atherogenesis processes (Shih et al., 2015; Canyelles et al., 2018). Prenatal arsenic treatment is reported to induce inflammation and accelerate AS by suppressing the activation of specific pathways required for gluconeogenesis and glycolysis (States et al., 2012). These dysbiotic functions in the GM metabolic pathways were affected following induction of CIH and might therefore associated with AS aggravation. There is a major limitation in this study. A study with antibiotics should be performed to substantiate the role of gut microbiota in this study. However, antibiotic administration would abolish pathogens and beneficial commensal microorganisms indiscriminately instead of specifically, which is of concern. Thus, consumption of antibiotics may further exacerbated microbiota dysbiosis through indulging the emergence of antibiotic-resistant bacteria. Future studies are urgently needed to substantiate the causal relationship between CIH aggravation AS and gut dysbiosis through optimizing the application of antibiotics.

In conclusion, the current study revealed specific microbial alterations caused in response to CIH during the development of AS, including similar but more potent shifts in fecal microbial composition and alterations in bacterial metabolic functions. The enhancement of *Halomonas*, *Halomonadaceae* and Oceanospirillales abundance and the reduction of *Sutterella* and in the gut were aggravated in AS mice by CIH administration. The microbial functions of replication recombination and repair proteins, glycan biosynthesis and metabolism, as well as metabolism of cofactors and vitamins may mediate the effect of CIH-induced GM dysbiosis in AS mice. By targeting gut bacteria, the development of OSA-associated metabolism dysfunction may be controlled.

## Data Availability Statement

The datasets presented in this study can be found in online repositories. The names of the repository/repositories and accession number(s) can be found below: NCBI, PRJEB42259.

## Ethics Statement

The animal study was reviewed and approved by Ethics Committee of Capital Medical University.

## Author Contributions 

JiL, YQ, CH and PW conceived the study, directed the project, designed the experiments and interpreted the results. CH, JiL and PW wrote the manuscript. YY and JuL performed the animal experiment model. XJ and HY performed histology analysis. JuL and CH analyzed the data. YQ, PW and YW revised the manuscript. All authors contributed to the article and approved the submitted version.

## Funding

This study was supported by the National Natural Science Foundation of China (Grant No. 81870335, 81670331, 81970224, 81500383, 81870308), the Beijing Natural Science Foundation (Grant No. 7192030, 7204242), Beijing Key Laboratory of Upper Airway Dysfunction and Related Cardiovascular Diseases (No: BZ0377), and the Beijing Hospitals Authority Youth Programme (code: QML20170303). The institutions had no role in the study design, data collection, data analysis and interpretation or writing of the manuscript.

## Conflict of Interest

The authors declare that the research was conducted in the absence of any commercial or financial relationships that could be construed as a potential conflict of interest.

## Supplementary Material

Supplementary Figure 1 Specific bacteria statistically significant between WT and AS, AS and AS+CIH. **(A, B)** Histogram of the LDA scores indicate bacteria differentially abundant between AS (red) and WT (green) at phylum **(A)** and genus **(B)** level. The LDA scores (log10) > 2 are listed. **(C, D)** LDA scores demonstrate phyla **(C)** and genera **(D)** that were significantly distinguished in AS mice (red) from the animals housed under CIH conditions (green). n=7 for WT, and n=8 for AS and AS+CIH.

## Abbreviation

OSA, obstructive sleep apnea; AS, arteriosclerosis; CIH, chronic intermittent hypoxia; apoE^-/-^, apolipoprotein E-deficient; HFD, high-fat diet; WT, wild-type; OUT, operational taxonomic units; GM, gut microbiota; PCoA, principal-coordinate analysis; NMDS, nonmetric dimensional scaling; LDA, Linear discriminant analysis; KEGG, Kyoto Encyclopedia of Genes and Genomes; PICRUSt, Phylogenetic Investigation of Communities by Reconstruction of Unobserved States.
