# Supplementary figures and images for: Chronic Intermittent Hypoxia Participates in the Pathogenesis of Atherosclerosis and Perturbs the Formation of Intestinal Microbiota
Source: Front Cell Infect Microbiol. 2021 Jul 1;11:560201. doi: 10.3389/fcimb.2021.560201 (PMC8281814; doi:10.3389/fcimb.2021.560201)

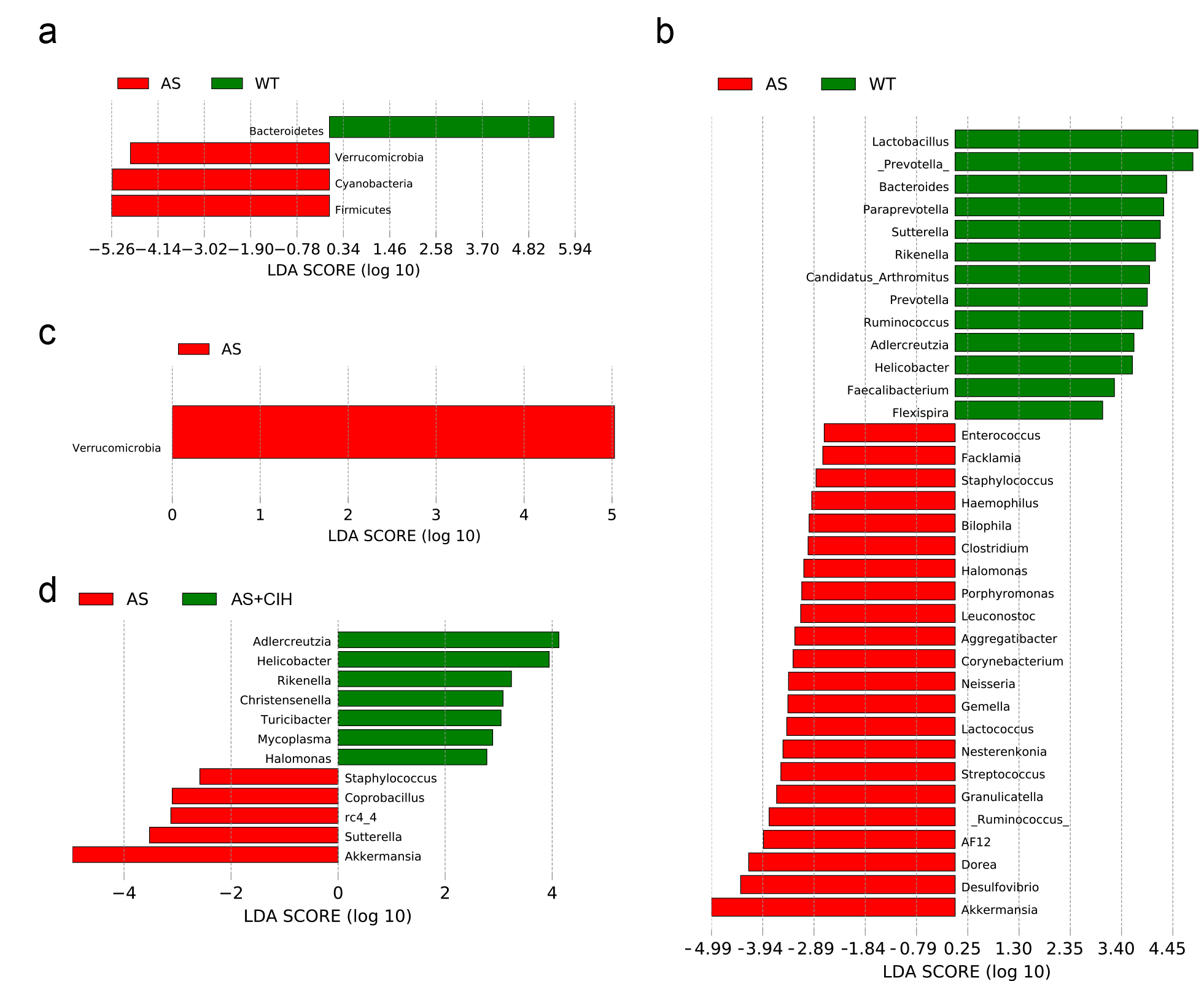

Supplement: Supplementary Figure 1 — Specific bacteria statistically significant between WT and AS, AS and AS+CIH. (A, B) Histogram of the LDA scores indicate bacteria differentially abundant between AS (red) and WT (green) at phylum (A) and genus (B) level. The LDA scores (log10) > 2 are listed. (C, D) LDA scores demonstrate phyla (C) and genera (D) that were significantly distinguished in AS mice (red) from the animals housed under CIH conditions (green). n=7 for WT, and n=8 for AS and AS+CIH. [file Image_1.tif]
